# Mechanical chest compressions for cardiac arrest in the cath-lab: when is it enough and who should go to extracorporeal cardio pulmonary resuscitation?

**DOI:** 10.1186/s12872-019-1108-1

**Published:** 2019-06-03

**Authors:** Bjarne Madsen Hardig, Karl B. Kern, Henrik Wagner

**Affiliations:** 1 Department of Cardiology, Lund University, 22242 Lund, Sweden; 2 Sarver Heart Center, University of Arizona, Rm. 005145, Tucson, AZ 85724 USA

**Keywords:** Cardiac arrest, Mechanical CPR, Cath-lab, PCI

## Abstract

**Background:**

Treating patients in cardiac arrest (CA) with mechanical chest compressions (MCC) during percutaneous coronary intervention (PCI) is now routine in many coronary catheterization laboratories (cath-lab) and more aggressive treatment modalities, including extracorporeal CPR are becoming more common. The cath-lab setting enables monitoring of vital physiological parameters and other clinical factors that can potentially guide the resuscitation effort. This retrospective analysis attempts to identify such factors associated with ROSC and survival.

**Methods:**

In thirty-five patients of which background data, drugs used during the resuscitation and the intervention, PCI result, post ROSC-treatment and physiologic data collected during CPR were compared for prediction of ROSC and survival.

**Results:**

Eighteen (51%) patients obtained ROSC and 9 (26%) patients survived with good neurological outcome. There was no difference between groups in regards of background data. Patients arriving in the cath-lab with ongoing resuscitation efforts had lower ROSC rate (22% vs 53%; *p* = 0.086) and no survivors (0% vs 50%, *p* = 0.001). CPR time also differentiated resuscitation outcomes (ROSC: 18 min vs No ROSC: 50 min; *p* = 0.007 and Survivors: 10 min vs No Survivors: 45 min; p = 0.001). Higher arterial diastolic blood pressure was associated with ROSC: 30 mmHg vs No ROSC: 19 mmHg; *p* = 0.012).

**Conclusion:**

Aortic diastolic pressure during CPR is the most predictive physiological parameter of resuscitation success. Ongoing CPR upon arrival at the cath-lab and continued MCC beyond 10–20 min in the cath-lab were both predictive of poor outcomes. These factors can potentially guide decisions regarding escalation and termination of resuscitation efforts.

## Background

Increasing interest to treat the underlying cause of cardiac arrest (CA) during resuscitation efforts has evolved since the first extended case series described the use of mechanical chest compressions (MCC) during refractory CA in the coronary catheterization laboratory (cath-lab) 8 years ago [1–5]. This treatment option has led to further studies treating refractory CA in the cath-lab setting [6]. Treatment with extracorporeal cardio pulmonary resuscitation (ECPR) has further developed this field, but also includes patients who experience CA outside the cath-lab setting, where early experience shows good outcome [4–7]. Some of these studies has been made as case series [5], ongoing randomized controlled trials (Prague NCT01511666), (Maastricht NCT03101787) (British Columbia NCT02832752) and other organizations have implemented this as a clinical routine [6]. In our early experience we observed that resuscitation in the cath-lab during simultaneous PCI vastly deviated from the normal ALS-algorithm recommended by guidelines. This demanded adjustments to the ALS-algorithms to be suitable for resuscitation efforts during simultaneous PCI, such as delaying further defibrillation attempts until coronary reperfusion is accomplished. These adjustments were recognized by resuscitation guidelines in 2015 [8]. The cath lab setting enables monitoring of physiological parameters and thus a more individual patient tailored treatment can be given [9]. Adequate levels of physiological parameters have previously been correlated with successful return of spontaneous circulation (ROSC) [7–13]. This retrospective analysis therefore explores multiple factors to identify which might be associated with ROSC and survival to understand which patients will do well with MCC alone and which may need ECPR.

## Methods

At the university hospital in Lund Sweden, a tertiary hospital with PCI facilities 24/7, 75 patients suffering CA in the cath-lab have been treated and evaluated in the cath-lab setting from January 1, 2004 to April 9, 2013 where outcome data that has been published [1, 5]. Detailed information regarding important clinical parameters were collected for 35 patients where, eight patients (23%) suffered CA out of hospital and arrived to the cath-lab with ongoing resuscitation efforts and 27 (77%) suffered CA during the intervention (with approval from the local ethical board in Lund (667/2009)). To evaluate the aspects of important confounding factors related to ROSC and survival, patients were divided into four different groups; data from patients that obtained ROSC were compared to data from those not obtaining ROSC and data for survivors were compared to non- survivors. The following data were collected and compared: Background data including concomitant diseases, reason to be admitted to the cath-lab i.e. the diagnosis, culprit lesion, rhythm causing the CA, drugs used during the resuscitation, anticoagulants used during the intervention, PCI-results, post ROSC-treatment and physiologic data collected during CPR. These data were compared for prediction of ROSC and survival differences.

## Statistics

Background parameters, resuscitation related parameters, PCI treatment variables and post ROSC treatment variables were presented as percentages for categorical data, while mean and standard deviation were used for continuous data, as appropriate. *P*-values for differences between the two groups was calculated using Fisher’s exact test for categorical data and using Mann-Whitney U test for numerical parameters. A P-value of < 0.01 was considered to indicate a significant difference as no other adjustment for multiple comparison was made.

## Results

**Fig. 1:**
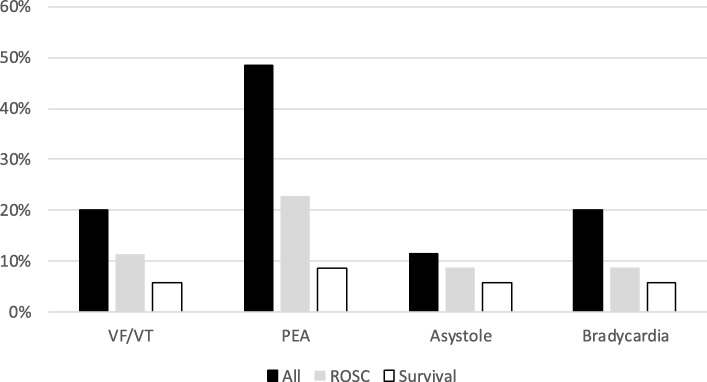
Shows the distribution of rhythm causing the arrest and its relation to ROSC and survival (VF/VT = ventricular fibrillation/ventricular tachycardia, PEA = pulseless electrical activity)

Eighteen (51%) of the patients gained ROSC and 9 (26%) patients survived with good neurological outcome. There was no difference between those that obtained ROSC and those that did not nor when comparing data from survivors and non-survivors in regards of background data including concomitant diseases, reason for admission to the cath-lab i.e. cardiac diagnosis, culprit lesion, rhythm causing the arrest (Table 1). Both ROSC patients and surviving patients had VF/VT (*N* = 7 (20%)), asystole (*N* = 4 (11%)), bradycardia (N = 7 (20%)) and pulseless electric activity (*N* = 17 (49%)) as the initiating rhythm for CA (Fig. 1). Fewer ROSC patients and surviving patients received epinephrine and the amount given was lower (Table 1). The CPR time was shorter for those that gained ROSC and survivors (Table 1). Those that arrived at the cath-lab with ongoing CPR had lower chance of obtaining ROSC (22% compared to 53% if the CA occurred in the cath-lab, not significant *P* = 0.086). None of the patients survived when resuscitation was ongoing when they were admitted to the cath-lab (Table 1). There was a significant higher median arterial early diastolic blood pressure among those that obtained ROSC compared to non-ROSC patients (30 (22–40) mmHg vs 19 (14–28) mmHg, *P* = 0.012), but only a trend to higher end diastolic and mean arterial pressures (Table 1), these numerical higher values were also seen among surviving patients however these were not significant (Table 1).

**Table 1:** Shows comparison of the data for patients that gained ROSC vs Non-ROSC patients as well for survivor’s vs Non-survivors

	All	ROSC	NO ROSC	P-value	Survivors	Non- survivors	*P*-value
	(*N* = 35)	(*N* = 18 (51%))	(N = 17 (49%))		(*N* = 9 (26%))	(*N* = 26 (74%))	
Background data (N (%) or Median (25th - 75th quartile))							
Gender (Females)	6 (17%)	3 (17%)	3 (17%)	> 0.99	1 (3%)	5 (19%)	> 0.99
Age (years)	72 (61–78)	71 (57–75)	76 (62–78)	0.306	74 (55–80)	71 (63–78)	0.806
AMI	10 (29%)	6 (33%)	4 (24%)	0.711	5 (56%)	5 (19%)	0.081
Angina pectoris	3 (9%)	3 (17%)	0 (0%)	0.229	0 (0%)	3 (12%)	0.553
Hypertension	18 (51%)	11 (61%)	7 (41%)	0.318	8 (89%)	10 (39%)	0.018
Diabetes	7 (20%)	3 (17%)	4 (24%)	0.691	3 (33%)	4 (15%)	0.340
Treated hypercholesterolemia	6 (17%)	3 (17%)	3 (18%)	> 0.99	2 (22%)	4 (15%)	0.635
Stroke	5 (14%)	4 (22%)	1 (6%)	0.338	1 (11%)	5 (19%)	> 0.99
Heart failure	3 (9%)	1 (6%)	2 (12%)	0.603	1 (11%)	2 (8%)	> 0.99
Asthma	2 (6%)	0 (0%)	2 (12%)	0.229	0 (0%)	2 (8%)	> 0.99
Renal disease	2 (6%)	1 (6%)	1 (6%)	> 0.99	0 (0%)	2 (8%)	> 0.99
Valvular disease	2 (6%)	0 (0%)	2 (11%)	0.299	0 (0%)	2 (8%)	> 0.99
Cancer	1 (3%)	0 (0%)	1 (6%)	0.486	0 (0%)	1 (4%)	> 0.99
Smoker	7 (20%)	4 (22%)	3 (18%)	> 0.99	2 (22%)	5 (19%)	> 0.99
X-Smoker	9 (26%)	5 (28%)	4 (24%)	> 0.99	4 (44%)	5 (19%)	0.192
Reason to be admitted to the Cath-lab (N (%))							
Planned Angiography	1 (3%)	1 (6%)	0 (0%)	> 0.99	0 (0%)	1 (4%)	> 0.99
Planned PCI	1 (3%)	1 (6%)	0 (0%)	> 0.99	0 (0%)	1 (4%)	> 0.99
N-STEMI	4 (11%)	3 (17%)	1 (6%)	0.603	2 (22%)	2 (8%)	0.268
STEMI (all)	26 (74%)	10 (56%)	16 (94%)	0.018	5 (55%)	21 (81%)	0.192
Inferior STEMI	9 (26%)	3 (17%)	6 (35%)	0.264	2 (22%)	7 (27%)	> 0.99
Anterior STEMI	16 (46%)	6 (33%)	10 (59%)	0.181	3 (33%)	13 (50%)	0.460
Lateral STEMI	1 (3%)	1 (6%)	0 (0%)	> 0.99	0 (0%)	1 (4%)	> 0.99
LBBB	1 (3%)	1 (6%)	0 (0%)	> 0.99	0 (0%)	1 (4%)	> 0.99
Acute stent occlusion.	1 (3%)	1 (6%)	0 (0%)	> 0.99	0 (0%)	1 (4%)	> 0.99
Tamponade	1 (3%)	0 (0%)	1 (6%)	0.486	0 (0%)	1 (4%)	> 0.99
Heart failure	1 (3%)	1 (6%)	0 (0%)	> 0.99	1 (11%)	0 (0%)	> 0.99
Culprit lesion (N (%))							
1 vessel	26 (74%)	12 (67%)	14 (82%)	0.433	5 (55%)	22 (85%)	0.162
2 vessels	7 (20%)	4 (22%)	3 (18%)	> 0.99	4 (44%)	3 (12%)	0.055
Left Main	12 (34%)	5 (28%)	7 (41%)	0.489	3 (33%)	9 (35%)	> 0.99
LAD	12 (34%)	6 (33%)	6 (35%)	> 0.99	3 (33%)	9 (35%)	> 0.99
M	2 (6%)	2 (11%)	0 (0%)	0.489	2 (22%)	0 (0%)	0.061
CX	4 (11%)	2 (11%)	2 (12%)	> 0.99	1 (11%)	3 (12%)	> 0.99
RCA	7 (6%)	1 (0%)	6 (12%)	0.229	1 (0%)	6 (8%)	> 0.99
Post lateral	1 (3%)	0 (0%)	1 (6%)	0.486	0 (0%)	1 (4%)	> 0.99
LIMA	2 (6%)	1 (6%)	1 (6%)	> 0.99	1 (11%)	1 (4%)	0.454
Rhythm causing the arrest (N (%))							
VF/VT	7 (20%)	4 (11%)	3 (9%)	> 0.99	2 (6%)	5 (14%)	> 0.99
PEA	17 (49%)	8 (23%)	9 (26%)	0.505	3 (9%)	14 (40%)	0.460
Asystole	4 (11%)	3 (9%)	1 (3%)	0.603	2 (6%)	2 (6%)	0.268
Bradycardia	7 (20%)	3 (9%)	4 (11%)	0.691	2 (6%)	5 (14%)	0.268
Drugs used during resuscitation efforts (N (%) or Median (25th - 75th quartile))							
Epinephrine injection (1 mg)	23 (66%)	9 (50%)	14 (82%)	0.075	2 (22%)	21 (81%)	0.003
Dose of Epinephrine	1 (0–3)	0 (0–1)	2 (2–5)	0.004	0 (0–0.5)	2 (1–4)	0.008
Nor-Adrenaline	24 (69%)	11 (61%)	13 (76%)	0.471	4 (11%)	20 (77%)	0.103
Atropine	11 (31%)	2 (11%)	9 (53%)	0.012	3 (33%)	8 (31%)	> 0.99
Amiodarone	1 (3%)	0 (0%)	1 (6%)	0.486	0 (0%)	1 (4%)	> 0.99
Dobutamine	11 (31%)	6 (33%)	5 (29%)	> 0.99	0 (0%)	11 (42%)	0.033
Levosimendan	1 (3%)	1 (6%)	0 (0%)	> 0.99	0 (0%)	1 (4%)	> 0.99
Isoprenaline	1 (3%)	0 (0%)	1 (6%)	0.486	0 (0%)	1 (4%)	> 0.99
Buffer	1 (3%)	0 (0%)	1 (6%)	0.486	0 (0%)	1 (4%)	> 0.99
Adenosine	1 (3%)	0 (0%)	1 (6%)	0.486	0 (0%)	1 (4%)	> 0.99
Anti-coagulants during PCI (N (%))							
Bivalirudin	19 (54%)	9 (50%)	10 (59%)	0.738	5 (55%)	14 (54%)	> 0.99
Klopidogrel	18 (51%)	10 (56%)	8 (47%)	0.740	5 (55%)	13 (50%)	> 0.99
Prasugrel	2 (6%)	2 (11%)	0 (0%)	0.489	2 (22%)	0 (0%)	0.061
Heparin	29 (83%)	15 (83%)	14 (82%)	> 0.99	8 (89%)	21 (81%)	> 0.99
Abciximab	3 (9%)	2 (11%)	1 (6%)	> 0.99	1 (11%)	2 (8%)	> 0.99
ASA	21 (60%)	11 (61%)	10 (56%)	0.733	6 (67%)	15 (58%)	0.712
Eptifibatide	1 (3%)	1 (6%)	0 (0%)	> 0.99	0 (0%)	1 (4%)	> 0.99
Fondaparinux	1 (3%)	1 (6%)	0 (0%)	> 0.99	0 (0%)	1 (4%)	> 0.99
Ticagrelor	4 (11%)	1 (6%)	3 (18%)	0.338	0 (0%)	4 (15%)	0.553
Warfarin	3 (9%)	2 (11%)	1 (6%)	0.658	1 (11%)	2 (8%)	> 0.99
PCI results (TIMI flow) (N (%))							
3	27 (77%)	14 (78%)	13 (72%)	> 0.99	7 (78%)	20 (77%)	> 0.99
2	1 (3%)	0 (0%)	1 (6%)	0.486	0 (0%)	1 (4%)	> 0.99
0	4 (11%)	1 (6%)	3 (17%)	0.338	1 (11%)	0 (0%)	> 0.99
CPR data (N (%))Median (25th - 75th quartile)							
CPR prior to arrival	13 (37%)	4 (22%)	9 (53%)	0.086	0 (0%)	13 (50%)	0.001
CPR during angiography	13 (37%)	5 (28%)	8 (47%)	0.305	3 (33%)	10 (38%)	> 0.99
CPR during PCI	26 (74%)	12 (67%)	14 (78%)	0.443	5 (55%)	21 (81%)	0.192
CPR time in the cath-lab	35 (12–52)	18 (10–41)	50 (33–60)	0.007	10 (8–25)	45 (30–60)	0.001
Post ROSC treatments (N (%) Median (25th - 75th quartile))							
Cooling	6 (17%)	6 (33%)	0 (0%)	0.019	1 (11%)	5 (19%)	> 0.99
Heartmate	1 (3%)	1 (6%)	0 (0%)	> 0.99	1 (11%)	0 (0%)	> 0.99
CABG	3 (9%)	3 (17%)	0 (0%)	0.229	1 (11%)	2 (8%)	> 0.99
Pacing	2 (6%)	0 (0%)	2 (12%)	0.229	0 (0%)	2 (8%)	> 0.99
IABP	12 (34%)	9 (50%)	3 (17%)	0.075	4 (44%)	8 (31%)	0.685
Heart transplantation	1 (3%)	1 (6%)	0 (0%)	> 0.99	1 (11%)	0 (0%)	> 0.99
Physiologic parameters during CA (Median (25th - 75th quartile))							
Arterial Systolic (mmHg)	85 (71–103)	85 (80–96)	84 (48–103)^a^	0.593	86 (77–98)	84 (70–103)^h^	0.494
Arterial Early Diastolic (mmHg)	24 (18–35)	30 (22–40)	19 (14–28)^b^	0.012	34 (23–40)	20 (14–30)^i^	0.050
Arterial End Diastolic (mmHg)	30 (21–44)	40 (24–46)	26 (18–29)^c^	0.071	41 (30–43)	27 (19–44)^j^	0.121
Arterial Mean (mmHg)	51 (41–58)	53 (48–59)	45 (32–58)^d^	0.074	54 (50–58)	46 (37–58)^k^	0.270
SpO_2_ (mmHg)	81 (74–86)	81 (73–82)	83 (74–89)^e^	0.599	77 (76–81)	82 (74–87)^l^	0.549
ETCO_2_ (mmHg)	21 (13–23)	21 (16–26)	21 (8–23)^f^	0.415	21 (15–26)	21 (13–23)^m^	0.653
LSCOt (%)	50 (44–56)	44 (44–44)	53 (45–57)^g^	NA	NA	48 (44–56)^n^	NA
RScot (%)	55 (49–58)	38 (38–38)	58 (55–59) ^g^	NA	NA	52 (49–58) ^n^	NA
AScot (%)	54 (48–57)	41 (41–41)	56 (54–57) ^g^	NA	NA	51 (48–57) ^n^	NA

*AMI* acute myocardial infarction, *PCI* percutaneous coronary intervention, *N-STEMI* non ST-elevation myocardial infarction, *STEMI* ST-elevation myocardial infarction, *LBBB* left bundle branch block, *LAD* left anterior descendent artery, *M* marginal branch, *Cx* circumflex artery, *RCA* right coronary artery, *LIMA* left internal mammary artery, *VF* ventricular fibrillation, *VT* ventricular tachycardia, *PEA* pulseless electrical activity, *SR* sinus rhythm, *ASA* acetyl salicylic acid, *CPR* cardio-pulmonary resuscitation, *CABG* coronary artery by-pass grafting, *IABP* intra aortic balloon counter pulsation,, *SpO*_*2*_ Index finger saturation, *ETCO*_*2*_ end tidal carbon dioxide, *LSctO*_*2*_ left cerebral tissue oximetry, *RSctO*_*2*_ left cerebral tissue oximetry, *ASctO*_*2*_ Average of left and right cerebral tissue oximetry and *NA* Not Applicable

^a^(ROSC *N* = 18 and No ROSC *N* = 16), ^b^ (ROSC *N* = 18 and No ROSC *N* = 15), ^c^(ROSC *N* = 18 and No ROSC *N* = 15), ^d^ (ROSC *N* = 14 and No ROSC *N* = 8), ^e^ (ROSC *N* = 18 and No ROSC *N* = 15), ^f^ (ROSC *N* = 7 and No ROSC *N* = 11), ^g^ (ROSC *N* = 1 and No ROSC *N* = 5), ^h^ (Survivors *N* = 9 and Dead *N* = 25), ^i^ (Survivors *N* = 7 and Dead *N* = 24), ^j^ (Survivors N = 7 and Dead N = 15), ^k^ (Survivors *N* = 8 and Dead *N* = 22), ^l^ (Survivors N = 7 and Dead N = 15), ^m^ (Survivors *N* = 4 and Dead N = 15) and ^n^ (Survivors *N* = 0 and Dead *N* = 6)

## Discussion

This analysis, although admittedly limited by the small number of patients, shows that it is possible to collect and monitor several important parameters that might be predictors of ROSC and survival during treatment of refractory CA patients in the cath-lab setting. The collected data generated a large amount of information from each patient in aspects of background data, concomitant diseases, cause of the CA, circulatory state, rhythm at the event of CA, type of chest compressions, drugs given during advanced life support (ALS), physiologic parameters during ALS, culprit vessel, PCI-outcome, ROSC and survival. From these data several useful parameters emerged, namely if the patient arrived at the cath-lab with ongoing CPR and the length of time MCC were required in the cath-lab to establish ROSC. Both these clinical factors were associated with ultimately poor outcomes. No patient survived who arrived at the cath-lab with ongoing CPR, while the median time of MCC in the cath-lab for those who did survive was 10 min versus 45 min for those patients not surviving.

Predictive parameters have been previously reported for cardiac arrest. These include co-morbid conditions (concomitant diseases, culprit vessel, circulatory state) that are known to affect outcome for STEMI and following PCI [14, 15] and the cause of the CA in these specific cases are often known (coronary artery occlusion) but also, parameters important for assessing CPR-quality [7, 14–16], parameters important for the assessment of blood pressure and perfusion that might be useful to predict ROSC and survival [10–13, 17, 18], factors as rhythm at the time of the CA [18], use of vasoactive drugs [19–22], duration of ALS, TIMI-flow and post-ROSC treatment [23], are also known factors that can affect outcome after CA and feasible to collect.

In this limited series of cath-lab cardiac arrest, initial rhythm did not predict outcome. One parameter reported in series of ECPR patients that was not included in our database is arterial lactate levels, which has appeared to be markedly different between survivors and non-survivors [21].

The median cath-lab CPR time among the non-surviving patients (45 min (30–60) in this series, is in the same range as that seen as unfavourable in some ECPR cohort reports. Lamhaut and collaborators showed a significant increase in survival when the decision point for ECPR was set at 20 min of ALS, from the prior 30 min [24]. This finding of a finite time limit of MCC in the cath-lab of 10 to 20 min to achieve good outcomes suggests that if such a time is approaching the decision for escalating therapy to ECPR must be made before it is too late. Prolonging the period of MCC support too long can impair the chances for a good outcome [1].

In the present case series, patients with both shockable and non-shockable rhythms achieved long-term survival with MCC (Fig. 1), whilst most studies using extracorporeal cardio pulmonary resuscitation have been restricted to patients in refractory VF/VT since these CA are assumed to be of a cardiac origin. However, our data shows that this assumption might not be correct and some patients with non-shockable rhythms may also respond to MCC while the cause of their cardiac arrest is treated in the cath lab.

## Limitation

The major limitation is the small number of patients in this case series. Further study with additional patients could better define other important factors regarding the usefulness of MCC in the cath-lab and which patients should go on to ECPR. Another way to overcome this limitation, we suggest collaboration with other cath-labs implementing similar treatment algorithms and monitoring to be able to collect a sufficient number of patients for these rare cases.

## Conclusion

When a cardiac arrest occurring in the cath-lab aortic pressure should be monitored during the resuscitation efforts striving for at least 30 mmHg in diastolic values. If this cannot be achieved escalation of therapy to ECPR should be considered. This decision should be made within the first 10–20 min of resuscitation efforts in the cath-lab, as longer periods are associated with a decrease in survival. Finally, cardiac arrest occurring before and still requiring resuscitation efforts upon arrival at the cath-lab should be considered for ECPR as continuing chest compressions alone resulted in poor outcome.

## Data Availability

The datasets used and/or analysed during the current study are available from the corresponding author and author HW on reasonable request.

## Abbreviations

ALS: Advanced life support
CA: Cardiac arrest
CPR: Cardio- pulmonary resuscitation
ECPR: Extracorporeal cardio pulmonary resuscitation
MCC: Mechanical chest compressions
PCI: Coronary catheterization laboratories
ROSC: Return of spontaneous circulation
TIMI: Thrombolysis in myocardial infarction
VF: Ventricular fibrillation
VT: Ventricular tachycardia
